# Rare partial trisomy and tetrasomy of 15q11-q13 associated with developmental delay and autism spectrum disorder

**DOI:** 10.1186/s13039-020-00489-z

**Published:** 2020-06-10

**Authors:** Yinghong Lu, Yi Liang, Sisi Ning, Guosheng Deng, Yuling Xie, Jujie Song, Na Zuo, Chunfeng Feng, Yunrong Qin

**Affiliations:** Department of Clinical Laboratory, Yulin Women and Children Care Hospital, Yulin, Guangxi Zhuang Autonomous Region 537000 People’s Republic of China

**Keywords:** 15q duplication related disorders, Partial trisomy and tetrasomy of 15q11-q13, Small supernumerary marker chromosomes, Copy number variation sequencing, Developmental delay

## Abstract

**Background:**

Small supernumerary marker chromosomes (sSMCs), are additional abnormal chromosomes, which can’t be detected accurately by banding cytogenetic analysis. Abnormal phenotypes were observed in about 30% of SMC carriers. Duplication of chromosome 15 and related disorders, characterized by hypotonia motor delays, autism spectrum disorder (ASD), intellectual disability, and epilepsy including infantile spasms, might be account for 50% of the total sSMCs.

**Case presentation:**

An 11-month-old infant with an sSMC found by banding cytogenetics was referred to our clinic because of developmental retardation and autism spectrum disorder. After several months of rehabilitation treatment, the progress of motor development was obvious, but the consciousness was still far from satisfied. High-resolution karyotype analysis, multiplex ligation-dependent probe amplification and copy number variation sequencing (CNV-Seq) were conducted to confirm the identity of the sSMC. A bisatellited dicentric sSMC was observed clearly in high-resolution karyotype analysis and a 10.16-Mb duplication of 15q11.1q13.2 (3.96 copies) together with a 1.84-Mb duplication of 15q13.2q13.3 (3 copies) was showed by CNV-Seq in the proband. It suggested that the molecular cytogenetic karyotype was 47,XY,+dic(15;15)(q13.2;q13.3). Furthermore, the clinical symptoms of the proband mostly fit 15q duplication related disorders which are characterized by hypotonia motor delays, autism spectrum disorder (ASD), and intellectual disability.

**Conclusion:**

We reported for the first time using CNV-Seq to detect sSMCs and find a partial trisomy and tetrasomy of 15q11-q13 associated with developmental delay and autism spectrum disorder. Our report indicates that CNV-seq is a useful and economical way for diagnosis of dup15q and related disorders.

## Background

Small supernumerary marker chromosomes (sSMCs), are extra abnormal chromosomes, which can’t be detected accurately by banding cytogenetic analysis. The incidence of sSMCs is 0.04% ~ 0.05% in live births [1–3]. Abnormal phenotypes were observed in approximately 30% of SMC carriers [4]. The effects on individuals with sSMCs can be varied, depending on the size of sSMCs and the level of mosaicism or other structure factors [4, 5]. Chromosome 15 might be account for 50% of the total sSMCs, of which 80% are present as an inverted duplication of 15 [2, 6].

While inverted duplication of proximal chromosome 15 (inv dup(15)) appearing in phenotypically normal individual chromosomes have been reported [7, 8], they are more tend to be result in mental retardation, structural malformation, behavioral problems and epilepsy [9–12], and rarely even psychosis or sudden unexplained death. 15q duplication syndrome (dup 15q) is caused by at least one extra fragment maternally derived copy of the Prader-Willi /Angelman within 15q11.2-q13.1 [13].

Here, we described a child with global developmental delay, who carried an sSMC from chromosome 15.

## Case presentation

An 11-month-old boy was referred to our clinic for genetic counseling of his developmental retardation. He was born to nonconsanguineous healthy parents at 39^+ 3^ weeks of gestation by natural delivery, with a birth weight of 2.7Kg. He was the second child of the family and his sister seemed no abnormal. His mother’s routine pregnancy test results were not abnormal, including that of B-ultrasound. The boy could not crawl or stand on his own, liked looking up, and seemed to be autistic tendencies even abnormal intelligence development. Neonatal diseases screening showed the levels within the normal range of Phenylketonuria/ thyroid stimulating hormone / Glucose-6-phosphate dehydrogenase / Congenital adrenal hyperplasia. The magnetic resonance imaging (MRI) result seemed to be normal and fit changes of his age. Video electroencephalogram detection showed abnormal result: total conductive β rhythm, slightly sharpened frontal waveform, poor sleep background. But, no clinical seizures such as convulsions were observed during the test. Liquid chromatography coupled to mass spectrometry using a quadrupole mass analyser analyse (LC-MS/MS) showed the level of ornithine and proline were slightly lower than the normal reference ranges. An unknown sSMC was found in his karyotype analysis. Whole-exome sequencing (WES) showed two unknown mutations: MED13L 20/30, CHR12: 116422120 NM_015335.4:c.4396C>T (p.Arg1466Cys); SOX3 1/1, ChrX: 139587110, NM_005634.2:c.116 C>T (p.Pro39Leu), which inherited from his mother.

## Methods

To confirm which chromosome the sSMC derived from, peripheral blood was collected from the family and a series of methods were conducted, including high-resolution karyotype analysis, multiplex ligation-dependent probe amplification (MLPA) and copy number variation sequencing (CNV-Seq). Single nucleotide polymorphism-array (SNP-array) was used as confirmatory tests to verify the copy number variations identified by CNV-Seq.

## Results

### High-resolution karyotype analysis

**Fig. 1:**
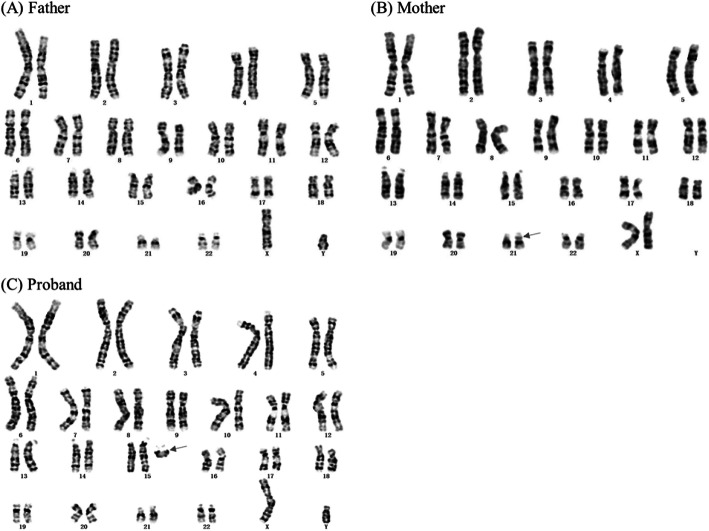
Three kinds of peripheral blood lymphocytes karyotypes of the proband and his parents. **a**: Karyotype analysis of the proband’s father showed no significant chromosomal abnormalities (46, XY). **b**: The karyotype of the proband’s mother was 46,XX,?inv(21)(p11q21). **c**: A bisatellited dicentric sSMC was observed in the proband. The arrows indicate the chromosomal defects

A bisatellited dicentric sSMC was observed clearly in high-resolution karyotype analysis of the proband, but which chromosome it came from still could not be confirm. His mother’s result was 46,XX,?inv(21)(p11q21). His father seemed no abnormal (Fig. 1).

### Multiplex ligation-dependent probe amplification (MLPA)

**Fig. 2:**
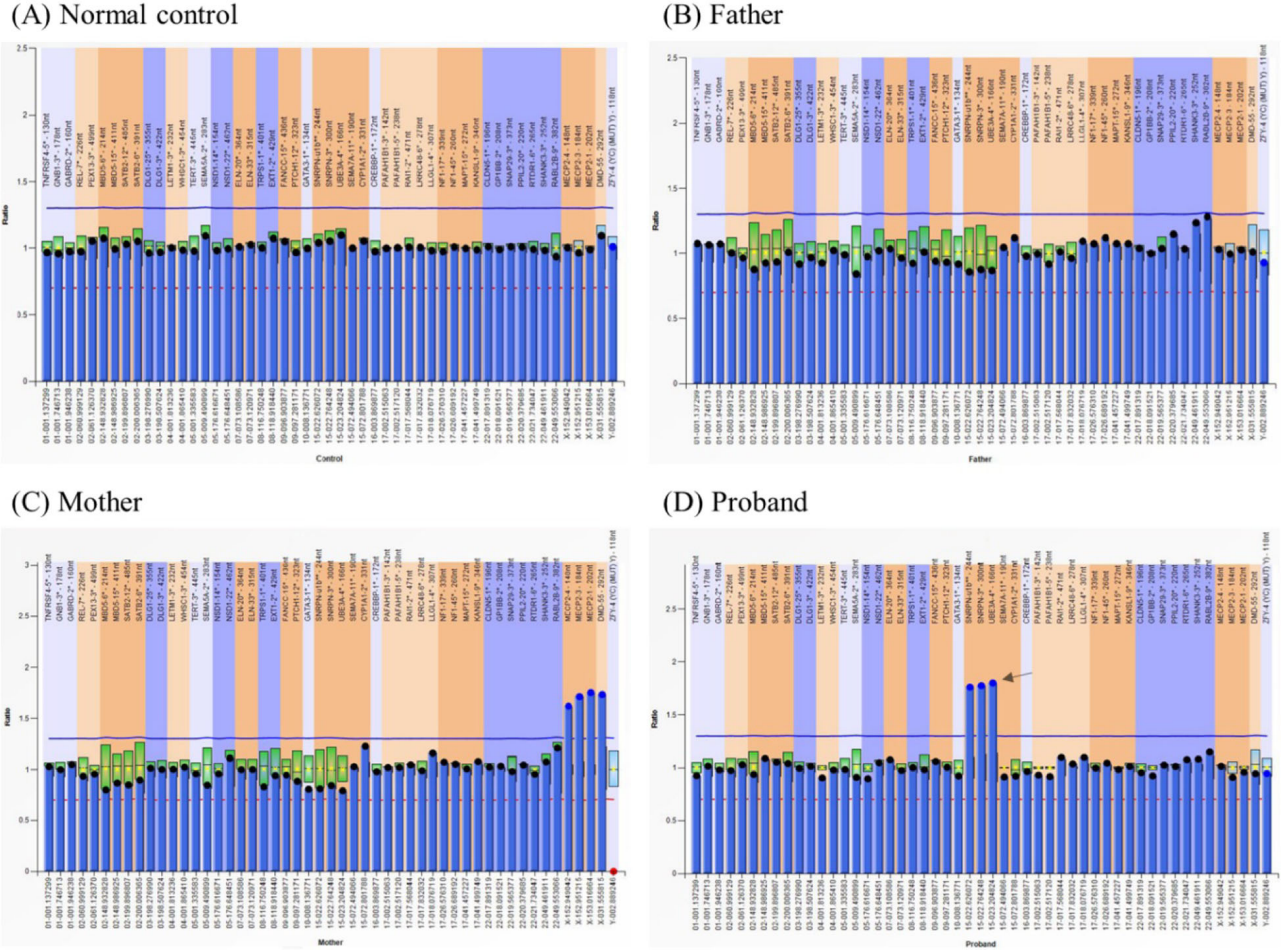
MLPA P245-B1 analysis of microdeletion syndromes. **a**: male normal control. **b**: father. **c**: mother. **d**: probes of 15q11.2 were more than two copies in the proband. The arrows indicate the chromosomal defects

Peripheral blood was collected to conduct MLPA with P245 probe mix (MRC-Holland) to find further information of the sSMC. Probes of 15q11.2 were more than two copies in the proband. But the results of his parents were normal (Fig. 2).

### CNV-Seq

**Fig. 3:**
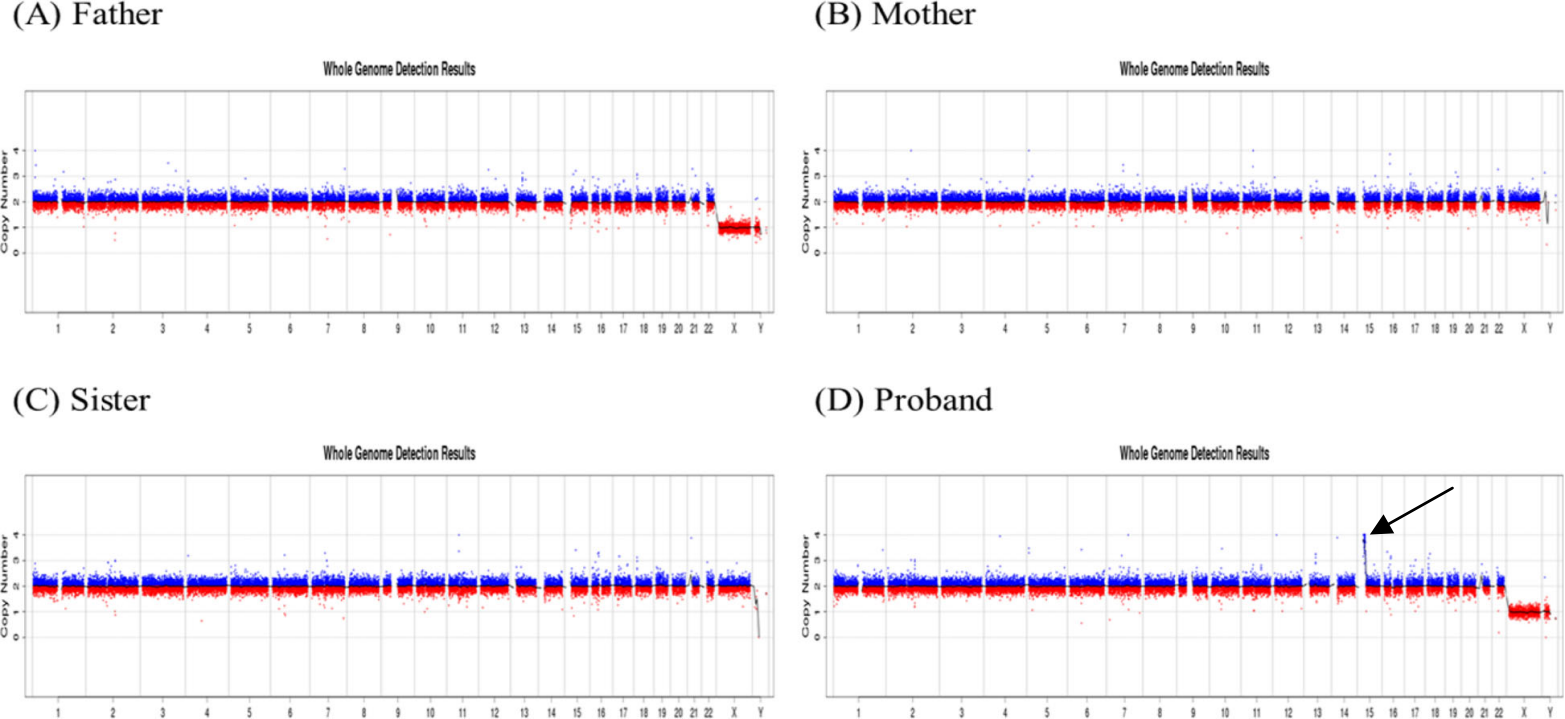
Whole genome detected by CNV-Seq in the family. **d**: CNV-Seq reveals an obvious de novo duplication of 15q in the proband. (arrow). **a**-**c**: There was no abnormal in the same position of the proband’s family member

**Fig. 4:**
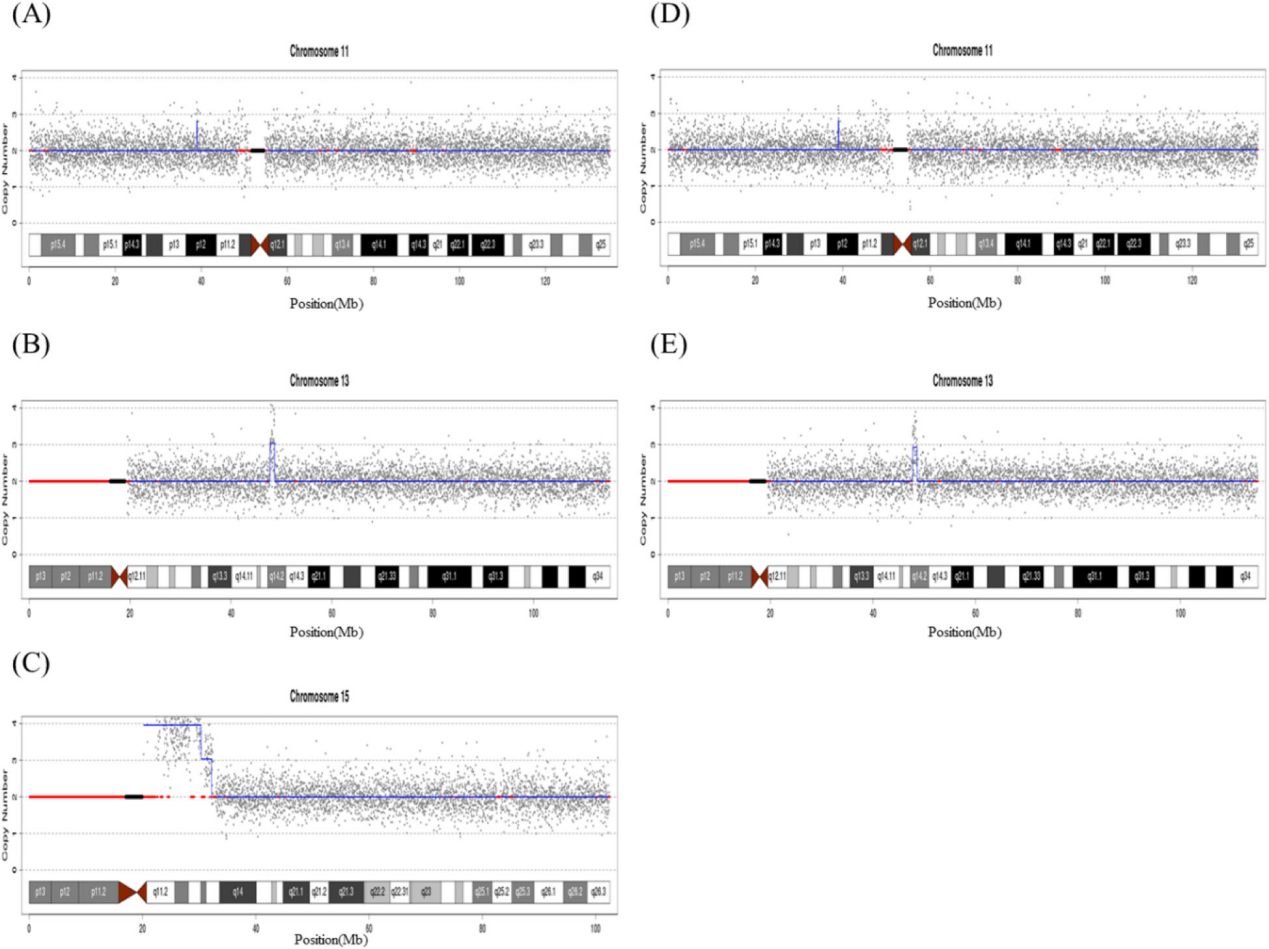
CNV-Seq analysis of the proband’s blood shows the result of dup(11)(p12)(38,940,000-39,080,000)(0.14Mb)(2.806);dup(13)(q14.2)(47,740,000-48,640,000)(0.90Mb)(3.039); dup(15)(q11.1-q13.2)(20,180,000-30,340,000)(10.16Mb)(3.96); dup(15)(q13.2-q13.3)(30,340,000-32,180,000)(1.84Mb)(3.034). **a**-**c**: Chromosome zoom-in view. Compared to the same positions of his mother (**d**) and father (**e**), the results of the proband showed a maternal origin of 11p duplication and a paternal origin 13q duplication

Peripheral blood was collected to conduct CNV-Seq and the result showed a 10.16-Mb duplication of 15q11.1q13.2 and a 1.84-Mb duplication of 15q13.2q13.3 in the proband: dup(15)(q11.1-q13.2)(20,180,000-30,340,000) (10.16 Mb) (3.96); dup(15)(q13.2-q13.3)(30,340,000-32,180,000) (1.84 Mb)(3.034); but the same results were not existing in his parents and sister. Furthermore, some unknown copy number variations were found in the proband: dup(11)(p12) (38,940,000-39,080,000) (0.14 Mb) (2.806); dup (13)(q14.2)(47,740,000-48,640,000) (0.90 Mb) (3.039); dup(16) (p11.2) (32,500,000-32,660,000) (0.16 Mb) (3.01); dup(18) (p11.31-p11.23) (7,060,000-7,560,000) (0.50 Mb) (2.887) (Figs. 3 and 4).

### SNP array

SNP array showed a 12.2-Mb duplication of 15q11.1q13.3, a 0.5-Mb duplication of 18p11.31p11.23 and a 1.04-Mb duplication of 13q14.2 in the proband; arr[hg19] 15q11.1q13.3(20,161,372-32,370,069)×4,18p11.31p11.23(7,070,642-7,573,510)×3,13q14.2(47,596,020-48,636,586) ×3.

## Discussion and conclusions

SSMCs occur in 0.044% of newborns, 0.075% of prenatal cases, 0.125% of subfertile cases and 0.288% of cases with mental retardation [14]. The majority of sSMCs are new mutations (about 70%), few cases are inherited from parents (about 30%) [15].

Most sSMCs are caused by a multiple-step mechanism: firstly, maternal meiotic nondisjunction, and then postzygotic anaphase lagging of the sSMC and its subsequent chromothripsis [16]. The sSMCs that originate from chromosome 15, commonly lead to 15q duplication syndrome (dup15q) and related disorders. Two mechanisms may be responsible for the cause of dup 15q: (1) about 20% of the cases are result from maternal interstitial 15q11.2-q13.1 duplication which generally includes one extra copy of 15q11.2-q13.1 within chromosome 15, resulting in trisomy for 15q11.2-q13.1; (2) about 80% of the cases are result from maternal isodicentric 15q11.2-q13.1 supernumerary chromosome – idic(15) – typically comprising two extra copies of 15q11.2-q13.1 and resulting in tetrasomy for 15q11.2-q13.1 [13].

Dup15q and related disorders are characterized by hypotonia motor delays, autism spectrum disorder (ASD), intellectual disability, and epilepsy including infantile spasms. Mostly, individuals with maternal idic (15) are more severely impacted than those with an interstitial duplication [13].

Dup15q and related disorders cannot be diagnosed accurately by conventional cytogenetic analysis. The extra copies of dup15q can be detected by molecular cytogenetic methods, such as the multiplex ligation-dependent probe amplification (MLPA), quantitative PCR (qPCR), chromosomal microarray analysis (CMA), fluorescence in situ hybridization (FISH) or other methods in previous literatures. In our research, copy number variation sequencing (CNV-Seq) was used to determine the copy number of sequences in the proband with an sSMC for the first time.

The results of high-resolution karyotype analysis (Fig. 1) and CNV-Seq of this proband and his parents showed that the child’s sSMC was new mutations (Fig. 3a, b, d). Before came to our clinic, an sSMC was found in the proband’s karyotype analysis, but further experiment should be taken to confirm the origin of the extra copies. First, high-resolution karyotype analysis was conducted and we found an sSMC with bisatellited dicentric feature which was similar to the mechanism of dup15q syndrome and related disorders (Fig. 1c). MLPA analysis showed that all 15q11.2 probes had a peak ratio higher than 1.5 within the PWS/AS region, which verified suppose that the sSMC may be derived from chromosome 15 (Fig. 1d). However, due to the limited coverage of the probes, the exact length and locus of the copies cannot be determined. The copy number, variation range, gene locus and fragment size of the 15q11–13 region were confirmed by CNV-Seq. SNP-array was used as confirmatory tests to verify the copy number variations (CNVs) identified by CNV-Seq. Similar results were obtained by SNP-array analysis. According to the results above, the sSMC was derived from chromosome 15 which is duplicated from end-to-end as a mirror image (idic (15)) (Table 1). Finally we suggested that the proband’s molecular cytogenetic karyotype is 47,XY,+dic(15;15)(q13.2;q13.3). However, if this case is not combined with CNV-Seq technology, only cytogenetic chromosome karyotype analysis could not find the microscopic abnormality of chromosome 15. Therefore, combination of multiple genetic methods for analysis is conducive to complementarity and verification.

**Table 1:** Genomic positions of variants identified in the SNP array and CNV-seq processed proband sample. Genomics coordinates are reported for human genome build hg19

Karyotype	SNP array positions			CNV-seq window boundaries			Prediction	Inherited from
	Start	Stop	Size (Mb)	Start	Stop	Size (Mb)		
11p duplication	N/A	N/A	N/A	38,940,000	39,080,000	0.14	Uncertain significance	Mother
13q duplication	47,596,020	48,636,586	1.04	47,740,000	48,640,000	0.9	Uncertain significance	Father
15q duplication	20,161,372	32,370,069	12.2	(1) 20,180,000	(1) 30,340,000	(1) 10.16	Pathogenic	De novo
				(2) 30,340,000	(2) 32,180,000	(2) 1.84	Pathogenic	De novo
16p duplication	N/A	N/A	N/A	32,500,000	32,660,000	0.16	benign	De novo
18p duplication	7,070,642	7,573,510	0.5	7,060,000	7,560,000	0.5	benign	Father

*Mb* Megabase; *N/A* not applied

It’s well known that CMA is one of the most commonly used methods to detect CNVs for decades. Today, CNV-seq is a new method to detect CNVs using the next-generation sequencing [17]. In our research, both CNV-seq and SNP array had find the origin of extra copies within the PWS/AS region in the proband. However, there were still some differences between the results in copy number of the two methods (see Table 1). CNV-Seq showed a 10.16-Mb duplication of 15q11.1q13.2, and a 1.84-Mb duplication of 15q13.2q13.3 in the proband: dup(15)(q11.1-q13.2)(20180000-30340000)(10.16Mb)(3.96);dup(15)(q13.2-q13.3)(30340000-32180000)(1.84Mb)(3.034). While SNP array presented a two copies duplication of 15q11.1q13.3: arr[hg19] 15q11.1q13.3(20,161,372-370,069) ×4. Reasons for this difference may be that the theories are different between these two technologies: SNP array is a microarray-based method while CNV-Seq is based on next-generation sequencing. The number of probes and DNA segments on a microarray does not represent the true copy number of sequences in an assembled genome. Especially, the regions including multiple copies are the hardest to assemble correctly which is still the key unsolved problem. Assembly errors such as these can lead to erroneous changes in sequencing coverage, resulting in erroneous indications of CNV [18]. On the contrary, sequencing-based methods, such as CNV-seq, are likely to obtain increased advantage over microarrays. Because next-generation sequencing technologies mostly produce a great deal of short reads and the number of reads sequenced, but not the length of the reads, is considered to be the most significant factor that determines the resolution, that is to say, larger number of sequenced pieces results in an increased resolution. Therefore, a large number of short reads, in a given constant resolution, is supposed to be an advantage rather than a small number of long reads [18]. In this means, compared to SNP array, CNV-seq may perform better in detecting Dup 15q syndrome. However, one advantage of SNP arrays over oligonucleotide arrays and CNV-seq is their ability to also detect uniparental disomy and consanguinity by the loss of heterozygosity of a series of consecutive SNP probes [19].

Effects on individuals with dup15q syndrome and related disorders can be varied, including hypotonia, autism spectrum disorder (ASD), intellectual disability, and infantile spasms [13]. Our patient seemed to be hypotonia motor delays, autistic tendencies and even abnormal intelligence development. When 11 months old, the boy could not crawl or stand on his own, paied no attention to any words of people even his parents. He didn’t even cry when being hungry or took blood from his veins.

The proband was born to nonconsanguineous parents who had given birth to a healthy girl before him. His mother’s routine pregnancy test results were not abnormal, including that of B-ultrasound. His mother had taken hypothyroidism drugs in early pregnancy, but there were no evidences show any relationship between hypothyroidism drugs and dup15q syndrome and related disorders. His mother worked in a subway station while his father traveled by air frequently because of the need of work. Whether the radiation caused by his parents’ jobs could lead to his congenital diseases needed further research.

Chen [20] had report a case with inv. dup (15), whose result was similarly to our case with the similar length and locus of the extra copies. But the specific starting and the end point were different. And in our research, CNV-Seq was first used to analyze inv. dup (15) or dup15q syndrome and related disorders. Symptoms in these two patients were similar, including developmental delay, hypotonia, poor speech, autism and intellectual. But no epilepsy or ataxia was found in our proband so far.

Another case [21] who carried the karyotype 47, XX, +inv. dup (15)(pter to q13:q13 was a girl with motor and mental retardation just like our case. But compared to our proband, the girl could sit at 8 months and stand at 18 months on her own. Moreover, the girl presented precocious puberty and epilepsy at about 8 years old, while whether these symptoms will appear in our patient needs to be followed up.

Li [22] had reported two patient suffered from sSMCs: one with a 15q partial octosomy (83%) another with a 15q partial hexasomy (72%). His research validated that the severity of phenotype was related to the mosaicism level and the dosage effect of the related genes in 15q.

Literatures about the treatment of dup15q syndrome and related disorders were mainly focus on epilepsy [23], few literatures focus on motor and mental retardation. It was reported that early postnatal OXT treatment could improve social abnormality in 15q dup mice [24]. Our patient had received several kinds of treatments from 11 months old, including mouse nerve growth factor (mNGF) treatment and physical therapy. The effects of mNGF therapy and physical therapy were not ideal. After several months of rehabilitation treatment, the progress of motor development was obvious, but the consciousness was still far from satisfied. At 19 months, it was assessed that the muscle tone was low, the development of gross and fine motor was backward, especially the development of intelligence. The proband received mouse nerve growth factor treatment and physiotherapy for 5 and 6 days, but the therapeutic effect was not obvious. After several months’ rehabilitation training, the sports progress was obvious, but the consciousness was still far from satisfactory. He could stand on his own for a few seconds in 22 months, but still couldn’t walk by 2 years old. Now the boy is receiving direct current therapy, we’ll keep track of the treatment.

Reviewing the experience of the proband before coming to our hospital, whole-exome sequencing (WES) was taken directly after an sSMC was found in the proband by karyotype analysis. Two mutations were found by WES: MED13L 20/30, CHR12: 116422120 NM_015335.4:c.4396C>T (p.Arg1466Cys); SOX3 1/1, ChrX: 139587110, NM_005634.2:c.116 C>T (p.Pro39Leu). The significance of both the mutations was not clear. The same mutations were found in his mother but not in his father. Obviously, WES method didn’t achieve the purpose of diagnosis to the proband, but did cause much cost to the family, furthermore, delay the patient’s golden treatment time. We suggest that, the identity of the sSMC but not the specific genetic mutations is the key problem which should be solved first. Choosing proper detection methods may shorten test period, and most importantly, reduce proband’s suffering and family cost, meanwhile the patient would receive symptomatic treatment timely.

In our research CNV-Seq was first used to detected sSMCs and dup15q syndrome and related disorders. We suggest that, when come across a marker chromosome, the origin of the sSMC rather than the specific genetic mutations is the key problem which should be solved first. Correct procedures in choosing detection technologies may shorten test period, and most importantly, reduce proband’s suffering and family cost, meanwhile the patient would receive symptomatic treatment timely. In this case, CNV-Seq may be a good choice.

It’s reported that people with 15q11-q13 dup syndrome or inv. dup (15) are pathogenic only when the extra parts inherited form mother, and the level of effect on brain function and development have much to do with the maternal gene dosage [25, 26]. Though CNV-Seq can be used in detecting dup15q syndrome and related disorders accurately, but it can’t determine whether the extra fragment is paternal or maternal without other technologies such as FISH or quantitative fluorescent polymerase chain reaction (QF-PCR).

## Data Availability

The datasets and material used or analysed during the current study are available from the corresponding author on reasonable request.

## Abbreviations

sSMCs: Small supernumerary marker chromosomes
CNV-Seq: Copy number variation sequencing
ASD: Autism spectrum disorder
WES: Whole-exome sequencing
MLPA: Multiplex ligation-dependent probe amplification
SNP-array: Single nucleotide polymorphism-array
